# A case of adenoid cystic carcinoma of the esophagus

**DOI:** 10.1186/s40792-015-0122-5

**Published:** 2015-11-27

**Authors:** Genta Sawada, Jeongho Moon, Akihisa Saito, Kazuki Odagiri, Yuri Kimura, Gen Takahashi, Shinya Yamashita, Masashi Inoue, Toshimitu Irei, Shin Nakahira, Yosuke Shimizu, Harumi Tominaga, Kazuya Kuraoka, Kiyomi Taniyama, Nobutaka Hatanaka

**Affiliations:** Department of Surgery, National Hospital Organization, Kure Medical Center, Chugoku Cancer Center, 3-1, Aoyamacho, Kure, 737-0023 Japan; Department of Diagnostic Pathology, National Hospital Organization, Kure Medical Center, Chugoku Cancer Center, Kure, Japan; National Hospital Organization, Kure Medical Center, Chugoku Cancer Center, Kure, Japan

**Keywords:** Adenoid cystic carcinoma, Esophageal cancer

## Abstract

Esophageal adenoid cystic carcinoma (EACC) is a very rare form of malignant tumor in the esophagus. Here, we report the case of a 78-year-old man who was diagnosed with EACC by preoperative endoscopic biopsy. Thoracoscopy-assisted subtotal esophagectomy with lymph node dissection was carried out. Microscopic examination of the resected specimen suggested that the tumor invaded to submucosal layer and showed no lymph node metastasis. Histologically, tumor primarily exhibited an alveolar solid pattern with partial cribriform and tubular patterns. Alcian blue staining showed many mucoid materials within the glandular cavity formed by tumor cells. Immunohistochemical studies revealed that the tumor cells reacted with pan-cytokeratin immunostains and expressed vimentin and S-100 protein. Collectively, the tumor was diagnosed as primary EACC, T1bN0M0 according to “Japanese Classification of Esophageal Cancer 10th edition.” The patient showed no recurrence sign 12 months after the surgery.

The current study also reviewed 35 EACC cases reported in Japanese literatures from 1990 to 2014. Combined with our case, we found that EACC is less frequently accompanied by lymph node metastasis as compared to esophageal squamous cell carcinoma, especially at the early stage. The prognosis of EACC is relatively better when tumors have no lymph node metastasis.

## Background

Esophageal adenoid cystic carcinoma (EACC) is a rare form of malignant tumor in the esophagus while relatively common in the parotid and salivary glands [1, 2]. Histologically, tumors are well known to consist of two main cell types: ductal and modified myoepithelial cells, and have three defined patterns: tubular, cribriform, and solid pattern. Characteristically, EACC is sometimes accompanied by squamous cell carcinoma (SCC) and basaloid squamous cell carcinoma (BSC) components, which can create different malignancy as adenoid cystic carcinoma (ACC) of the parotid and salivary glands. In contrast to these histological features, little information is available on clinical features and management because the number of case reports on EACC is limited. With regard to the prognosis, previous studies demonstrated conflicting findings: Some studies explained that, as compared to SCC, frequent lymph node metastasis and vascular invasion provided patients with poor prognosis [3, 4]. On the other hand, other studies recently reported that lymph node metastasis occurred less frequently at the early stage and prognosis was better than that of SCC. Ishii et al. reported that the average overall survival of EACC was 25 months when the tumor had no mixture of SCC or BSC component [5].

Here, we report a case of primary EACC without metastasis that underwent thoracoscopy-assisted subtotal esophagectomy with lymph node dissection. In addition, to disclose the clinical features and resolve conflicting results, we presented a summary of 35 cases of EACC reported from 1990 to 2014 in Japanese literatures.

## Case presentation

A 78-year-old man was admitted for the treatment of gastric ulcer in our hospital. He was a heavy smoker without habitual drinking, and presented no lymphadenopathy in the cervical or supraclavicular regions. Laboratory examination suggested no remarkable findings, and his serum levels of carcinoembryonic antigen, carbohydrate antigen 19-9, and squamous cell carcinoma related antigen were 0.8 ng/ml, 9.0 U/ml, and 1.6 ng/ml, respectively.

**Fig. 1:**
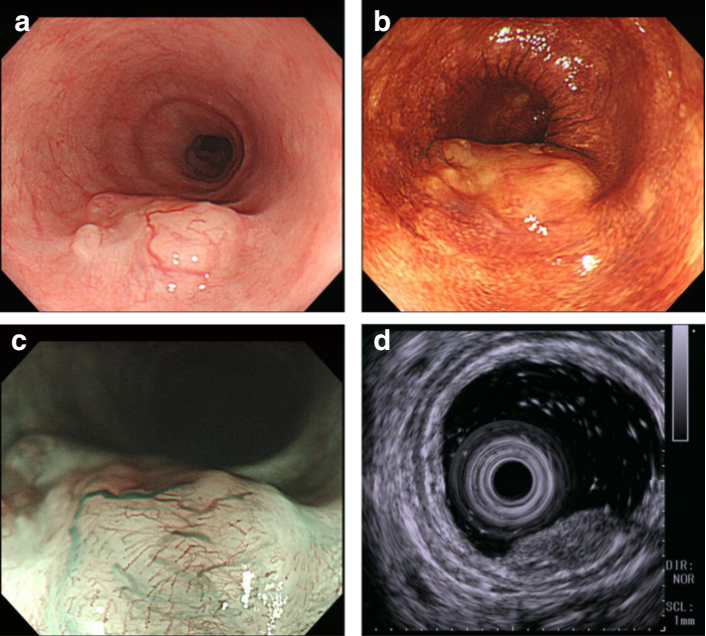
Endoscopic findings. **a** Protruding lesion located in the middle of the esophagus. **b** The tumor region was faintly stained with iodine. **c** Narrow-band imaging of the lesion suggested irregularity of intraepithelial papillary capillary loops. **d** Endoscopic ultrasonography detected a mixed echogenic lesion in the submucosal layer

**Fig. 2:**
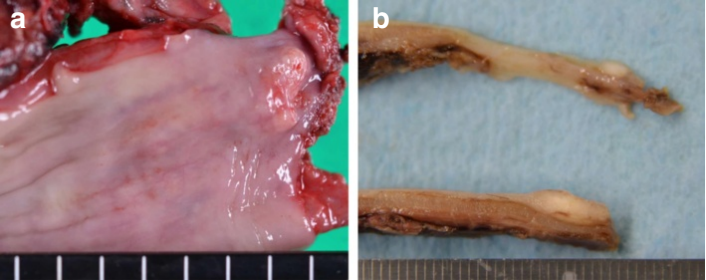
Resected tissue specimen. **a** The solid mass measuring 1.0 × 0.7 cm in size. **b** Solid mass with graywhite appearance locating mainly in the submucosal layer was found on the cut surface of the tumor

**Fig. 3:**
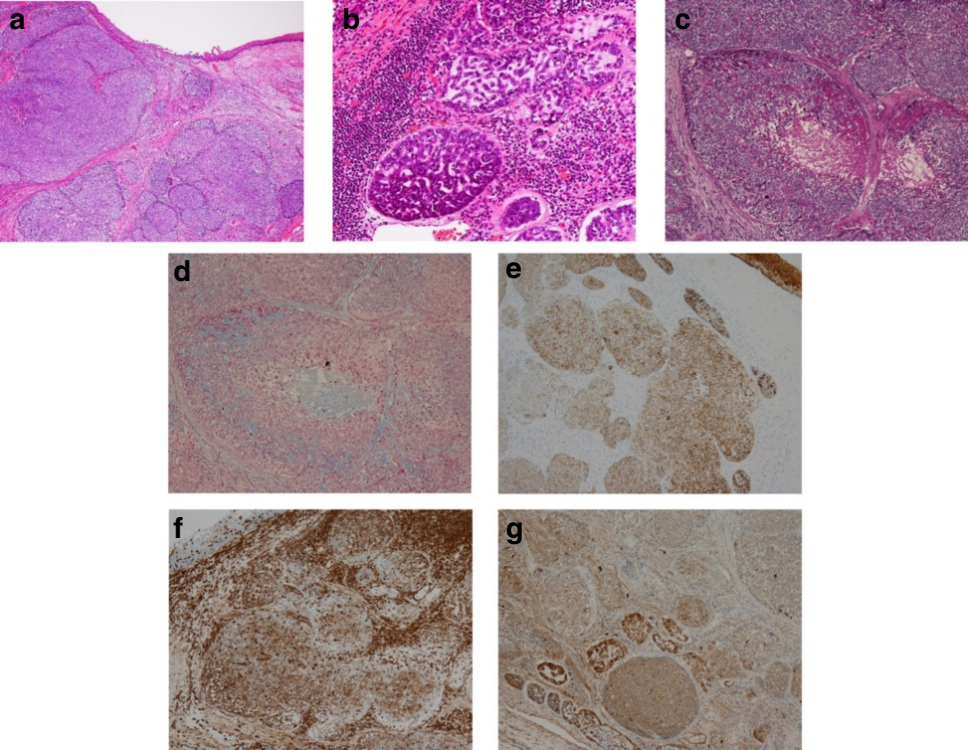
Histological examination. **a**, **b** Microscopic examinations showed alveolar solid and cribriform patterns in the resected specimen (hematoxylin and eosin staining ×40, 200). **c** PAS and **d** Alcian blue staining of intraluminal substance in the tubular pattern region (×100). **e**–**g** Immunohistochemical staining with **e** cytokeratin, **f** vimentin, and **g** S-100 protein (×100)

Endoscopic survey for the follow-up of gastric ulcer revealed a protruding lesion with smooth surface and central excavation located in the middle of the esophagus (Fig. 1a). Iodine stain showed unambiguous lightly stained region (Fig. 1b), which suggested that the tumor was covered by normal esophageal mucosa. Irregular patterns of intra-epithelial papillary capillary loops were found by narrow-band imaging (Fig. 1c), and lesions with mixed echogenicity in the submucosal layer were detected by endoscopic ultrasonography (Fig. 1d). Although the results of ordinary biopsy suggested chronic esophagitis in the mucosa, a boring biopsy at the following endoscopic survey allowed us to observe a mixture of cribriform and solid patterns in the tumor component, which strongly indicated ACC in the esophagus. Computer tomography could not detect any primary lesion in the esophagus and found no lymph node or distant metastasis. Therefore, under the diagnosis of primary EACC without metastasis, a thoracoscopy-assisted subtotal esophagectomy with lymph node dissection was carried out, followed by posterior mediastinal gastroesophagostomy. The resected tissue specimen showed a non-encapsulated solid mass with slight erosion, measuring 1.0 × 0.7 cm in size (Fig. 2a). The cut surface of the tumor demonstrated a solid mass gray-white appearance mainly located in the submucosal layer (Fig. 2b). Microscopic examination found no tumor invasion to the proper muscle layer without lymph node metastasis (pT1bN0M0 stage I according to “Japanese Classification of Esophageal Cancer 10th edition”). An alveolar solid pattern was predominantly shown (Fig. 3a) while cribriform and tubular patterns were partially identifiable in the lesion (Fig. 3b). Intraluminal substance in the tubular pattern regions was stained with alcian blue and periodic acid-Schiff (PAS) (Fig. 3c, d), which indicated that the component contained basophilic glycosaminoglycans and hyalinized basal lamina material. Immunohistochemistry revealed that the tumor cells reacted with pan-cytokeratin immunostains (Fig. 3e) and expressed vimentin and S-100 protein (Fig. 3f, g), collectively supporting the pathological diagnosis of primary EACC. The patient was discharged 63 days after operation, and no sign of recurrence was detected in the 12 months of follow-up.

### Discussion

The incidence of EACC was identified with 0.04 % (1 of 2370 cases) and 0.16 % (4 of 2521 cases) of esophageal malignant tumors by Igaki et al. [6]. and Hosokawa et al. [7], respectively. Using Japan Medical Abstracts Society (http://login.jamas.or.jp/) to search with the keywords “esophagus” and “adenoid cystic carcinoma”, we found 35 case reports on EACC from 1990 to 2014 (hereafter, a combination of these cases and our case are referred to as the reviewed cases) (Table 1). The average age of the reviewed cases was 66.4 years, and the sex ratio was 30 men to 6 women. The most prevalent tumor appearance in the endoscopic findings was protruded (58.6 %), and the second was ulcerative (24.1 %). This was consistent with previous reports which concluded that EACC was derived from esophageal glands, as the tumor was usually covered by normal mucosa at the early stage and ulceration was subsequently exhibited as the tumor progressed [8, 9].

**Table 1:** Clinical factors of the current case and 35 reviewed cases of EACC reported in Japan from 1990 to 2014

Year	Age/sex	Endoscopic appearance	Biopsy	Treatment	Depth of invasion	Lymph node metastasis	Lymphatic invasion	Vascular invasion	Observation period (month)	Outcome (cause of death)
Present	78/M	Protruding	ACC	Surgery	SM	−	−	−	2	Alive
1995	74/M	Elevated	ACC	Surgery	SM	−	+	+	12	Alive
1991	70/M	Protruding	ACC	Surgery	SM	−	−	+	N.A	N.A
1995	59/M	Protruding	ACC	Surgery	SM	−	−	−	13	Alive
2011	67/F	Protruding	ACC	Surgery	SM	−	−	−	N.A	N.A
2012	64/M	Protruding	ACC	Surgery	SM	−	−	−	N.A	N.A
2005	81/M	N.A	N.A	Surgery	SM	−	N.A	N.A	69	Dead (other disease)
2005	69//M	N.A	N.A	Surgery	SM	−	N.A	N.A	51	Alive
1997	53/M	Elevated	SCC	Surgery	SM	−	+	+	36	Alive
1994	81/M	Ulcerative	SCC	Surgery	SM	−	−	−	26	Alive
2001	71/M	Protruding	SCC	Surgery	SM	−	−	−	41	Alive
2010	60/M	Protruding	SCC	NAC + surgery	SM	−	−	−	5	Alive
1997	81/M	N.A	N.A	Surgery	SM	−	−	−	22	Alive
2003	65/M	Protruding	N.A	Surgery	SM	−	−	+	4	Dead (other disease)
1990	74/M	Protruding	SCC	Surgery	SM	−	−	−	30	Alive
1992	70/F	Protruding	N.A	Surgery	SM	−	−	−	142	Alive
1990	69/M	Protruding	SCC	Surgery	SM	−	−	−	6	Alive
1990	73/M	Protruding	SCC	Surgery	SM	−	−	−	18	Alive
1998	48/F	Ulcerative	ACC	Surgery	MP	+	+	+	36	Alive
1996	57/M	N.A	N.A	Chemotherapy + surgery	MP	−	+	+	56	Alive
2005	68/M	N.A	N.A	Surgery	MP	−	N.A	N.A	2	Alive
1994	59/M	Elevated	SCC	Surgery	MP	−	+	−	60	Alive
1991	64/M	Ulcerative	SCC	Surgery	MP	−	−	−	11	Alive
1992	59/M	Ulcerative	SCC	Surgery	MP	−	−	+	42	Alive
1996	79/M	Protruding	Adeno	Surgery	MP	−	N.A	N.A	30	Alive
1994	61/M	Ulcerative	ACC	Surgery	AD	N.A	−	+	12	Dead (EACC)
2007	51/M	Ulcerative	ACC	Surgery	AD	−	−	+	2	Alive
1994	71/M	Protruding	ACC	Surgery	AD	+	+	+	5	Dead (other disease)
2005	41/M	N.A	N.A	Radiation	AD	-	N.A	N.A	22	Dead (EACC)
1991	57/M	Elevated	SCC	Surgery	AD	+	N.A	N.A	N.A	N.A
1996	68/F	Protruding	SCC	Surgery	AD	−	N.A	N.A	N.A	N.A
1992	64/M	Elevated	N.A	Surgery	AD	+	−	+	15	Dead (EACC)
1993	77/F	Protruding	ACC	Chemotherapy	AI (aorta)	+	N.A	N.A	9	Dead (EACC)
1992	55/M	Ulcerative	SCC	Surgery	AI (trachea)	+	+	+	7	Dead (EACC)
2005	84/M	N.A	N.A	Surgery	AI	+	N.A	N.A	40	Alive
1997	70/F	Protruding	SCC	Surgery	N.A	+	N.A	N.A	30	Dead (other disease)

*N.A* not available, *adeno* adenocarcinoma, *NAC* neoadjuvant therapy, *SM* submucosa, *MP* muscle, *AD* adventitia, *AI* invasion to adjacent organ

For preoperative diagnosis of EACC, it has so far been reported that endoscopic biopsy frequently failed to provide a correct diagnosis of EACC because of its similar growth pattern like submucosal tumor. In addition, accurate diagnosis may be more challenging when EACC contained SCC or BSC components [10]. Morisaki et al. reported that only 8 of 37 cases (21.4 %) in and before 1996 were accurately diagnosed as EACC with endoscopic biopsy specimens [11]. In the reviewed cases, correct diagnosis was shown in 11 of 26 cases (43.2 %) (diagnosis with biopsy specimen was available for 26 of 36 cases). These results indicated an improvement in diagnosis accuracy, which was probably made possible by the development of endoscopic devices such as boring biopsy and narrow-band imaging system. Another factor is the establishment of useful markers such as vimentin and S-100, which help to differentiate EACC from BSC diagnosis [12, 13].

The average overall survival of the reviewed cases was 27.6 months following clinical diagnosis. At the time of report, 22 cases were alive while 5 cases died of the EACC (Table 1). Half of the reviewed cases showed tumor invasion to the submucosal layer, indicating the advancement of early EACC detection. Moreover, out of 25 cases with the tumor invading to the submucosal layer or muscularis propria, only one case showed lymph node metastasis and no subjects died of the EACC. These results suggested that early detection of the tumor provides patients with the possibility of a complete cure. For treatment, radical resection with regional lymph node dissection is considered to be the first option, since a susceptibility for chemotherapy and radiation therapy still remains elusive [14–16]. In the reviewed cases, 34 cases underwent radical resection, of which one had neoadjuvant chemotherapy consisting of low-dose CDDP and 5-FU, and another one was treated with intensive repeated combination chemotherapy with CDDP and 5-FU for 1.3 years before surgery. We found only one case that underwent combination chemotherapy with etoposide and tegafur for the treatment of liver metastasis of EACC. Intriguingly, there was a case without metastasis that received radiation therapy alone and subsequently survived for 22 months.

In the current case, we were able to apply thoracoscopy-assisted surgery to this patient because the depth of tumor invasion was diagnosed as submucosa before the surgery. Concomitant with other reviewed cases, our case showed no lymph node metastasis. We hence expected that the subsequent prognosis of this patient may be good.

## Conclusions

Although the number of the reviewed cases who are observed in a long period is limited, we considered that prognosis of EACC is relatively better if the tumor is detected with no lymph node metastasis. The incidence of lymph node metastasis of EACC is less frequent as compared to that of SCC, especially when the tumor invades to the submucosal layer or muscularis propria. Therefore, less invasive surgery such as thoracoscopic surgery may be suitable for EACC at the early stage. Moreover, previous study reported that a case with EACC in submucosa was successfully treated by incisional endoscopic enucleation [17]. For applying the endoscopic treatment practically, we need large scale study on EACC to make a sufficient evaluation for the probability of lymph node metastasis as well as lymphatic and vascular invasion.

## Consent

Written informed consent was obtained from the patient for publication of this Case Report and any accompanying images. A copy of the written consent is available for review by the Editor-in-Chief of this journal.

## Abbreviations

ACC: adenoid cystic carcinoma
BSC: basaloid squamous cell carcinoma
EACC: esophageal adenoid cystic carcinoma
SCC: squamous cell carcinoma
